# The Effects of Extrinsic Values on Unethical Decision Making and Behaviour

**DOI:** 10.3390/bs15111479

**Published:** 2025-10-30

**Authors:** Paton Pak Chun Yam, Su Lu, Allan B. I. Bernardo, Lisbeth Ku

**Affiliations:** 1 Faculty of Health and Life Sciences, De Montfort University, The Gateway, Leicester LE1 9BH, UK; paton.yam@dmu.ac.uk (P.P.C.Y.); su.lu@dmu.ac.uk (S.L.); 2 Department of Psychology, De La Salle University, 2401 Taft Avenue, Manila 1004, Philippines; allan.bernardo@dlsu.edu.ph

**Keywords:** value orientation, unethical behaviour, cross-cultural research, experimental manipulation, self-determination theory

## Abstract

Engaging in unethical behaviours, such as cheating, lying, stealing and fraud, holds significant consequences for individuals and the broader community. Drawing on self-determination theory, we posit that in a consumer-centric society, where one’s worth is often linked to wealth, celebrity status, and appearance, individuals who adopt extrinsic values might be motivated to engage in unethical behaviour in pursuit of financial gains. Study 1 surveyed university students in Macao, China (*n* = 566), and crowdsourcing workers from the UK (*n* = 605), demonstrating that extrinsic values were linked to unethical decision-making in vignette-based scenarios. This association was held in both societies, suggesting a culture-independent connection between unethicality and values. To establish causal relationships, we conducted experiments manipulating extrinsic cues participants received in Macanese (Study 2, *n* = 170) and British (Study 3, *n* = 197) participants. Results revealed a significant impact of these cues on behavioural measures of unethicality, with those in the extrinsic-cues condition more likely to lie for financial gains compared to those in the control groups. Together, these findings highlight the influence of extrinsic values on unethical behaviour across cultural contexts. This research underscores the urgent need to address societal norms and consumerist cues that focus on extrinsic values, which may erode ethical standards and threaten collective well-being.

## 1. Introduction

Over the past few decades, corporate and financial frauds have become a recurring story in the media. A significant instance involves the conviction of fraud and money laundering of Sam Bankman-Fried, renowned as the ‘crypto-king,’ who previously operated the world’s largest cryptocurrency exchange and possessed an estimated fortune of US$26 billion (Sherman & Hoskins, 2023). The academia, too, has not been immune from scandal. One of the most recent incidents involves allegations of data fraud against two of the world’s leading researchers who study dishonesty (Lewis-Krausk, 2023). Although these instances, while sensational, might seem like isolated events involving a handful of individuals, there is evidence to suggest a connection between an individual’s behaviour and the social, economic, and cultural environment in which they reside. Indeed, findings from a study spanning 40 countries indicate a strong correlation between cheating on exams and the perceived level of corruption at the country level (Orosz et al., 2018). As the first step to address this societal issue, this paper examines how the endorsement of certain values by individuals may lead to unethical behaviours.

In psychology, unethical behaviour is often defined as actions that are ‘either illegal or morally unacceptable to the larger community’ (Jones, 1991, p. 367). In the past two decades, there has been an upsurge of interest in the when and why people engage in unethical behaviour (see Gerlach et al., 2019; Jacobsen et al., 2018, for reviews). Concurringly, there are calls for a deeper understanding of the motivational underpinning of unethicality through an overarching, unifying approach (e.g., Fleeson et al., 2022; Jacobsen et al., 2018).

In response to these calls, the present research proposes that diverse forms of unethical conduct stem from societal values internalised by individuals. This notion is underscored by Kasser et al.’s (2007) discussion on American Corporate Capitalism (ACC) (Kasser et al., 2007), highlighting its promotion of self-interest-centred values such as financial success, consumption, and competition. In evaluating Kasser et al.’s assertion, Schwartz (2007) examined individual values prevalent in 20 countries that adopt varying forms of capitalism, from competitive economies like ACC, where market competition serves as the primary coordination mechanism, to more collaborative economies seen in countries like Austria and Germany. Evidence suggests that individuals in competitive, market-driven societies exhibit a stronger preference for hierarchy and prioritise self-enhancement values over self-transcendence values, compared to individuals in societies with more collaborative economies (Schwartz, 2007).

These findings suggest that values endorsed by a society can be internalised by individuals and guide their motivation (Sagiv & Roccas, 2021). Given that many societies promote a culture centred around money and consumption, factors seen as integral to a “good life” (Kasser, 2015), we propose that extrinsic values-defined as the pursuit of personal wealth, adherence to a narrowly defined beauty standard, and the quest for power and status among peers (Kasser & Ryan, 1993, 1996)-could be the underlying drivers of various forms of unethical behaviour. To understand this relation, we turn to the self-determination theory (SDT; Ryan & Deci, 2017, 2019) as the guiding theoretical framework for our investigation.

SDT is an empirically supported meta-theory of human motivation comprising six interrelated “mini-theories,” each addressing a different aspect of motivation and psychological functioning. The current research is primarily informed by Goal Contents Theory (GCT; Kasser & Ryan, 1993), which focuses on the *“what”* of motivation—namely, the content of individuals’ goals and values. GCT distinguishes between extrinsic and intrinsic aspirations and their consequences for individual, social, and societal outcomes (Ryan & Vansteenkiste, 2023). A substantial body of cross-cultural evidence shows that extrinsic values are consistently associated with need frustration and ill-being, whereas intrinsic values promote need satisfaction and a range of positive outcomes (e.g., Bradshaw et al., 2023). By applying SDT and GCT to the study of unethical decision-making, the present research seeks to extend this theoretical tradition to explain how prioritising extrinsic goals may motivate individuals toward unethical conduct, regardless of cultural background or experimental context.

It is important to note that within the SDT framework, extrinsic values are frequently described as materialistic in nature (e.g., Kasser et al., 2004, p. 13; Kasser, 2016, pp. 491–492; Vansteenkiste et al., 2006, p. 2892). Indeed, Kasser et al. (2004) described a materialistic value orientation as “the belief that it is important to pursue the culturally sanctioned goals of attaining financial success, having nice possessions, having the right image (produced, in large part, through consumer goods), and having a high status (defined mostly by the size of one’s pocketbook and the scope of one’s possessions)” (p. 13). The domains mentioned in this definition of materialistic values (money, image, status/popularity) correspond to the extrinsic values of the Aspiration Index (AI), a measure developed by Kasser and Ryan (1996) that have been frequently used in the literature to measure *both* extrinsic values and materialistic values (see Dittmar et al., 2014). All these demonstrate how the terms “extrinsic values” and “materialistic values” refer to the same constructs within the SDT tradition, despite materialism has also been defined more narrowly in pertinent research (e.g., Richins & Dawson, 1992). We will therefore use the two terms interchangeably throughout this paper and reference relevant research where appropriate.

Why may there be a link between extrinsic/materialistic values and unethicality? According to GCT, extrinsic values crowd out intrinsic aspirations due to limited resources to attain both ideals (Kasser & Ryan, 2001). They instead motivate behaviours that depend not on the enjoyment for their own, but on external contingencies of rewards and punishments (Ryan & Deci, 2017; Vansteenkiste et al., 2006). The pursuit of extrinsic rewards thwarts one’s sense of autonomy and in turn makes one become vulnerable to basic psychological need frustration and dissatisfaction (Niemiec et al., 2009). To compensate, those who are extrinsically aspirated may prioritise short-term, risky, or antisocial means to ends over long-term growth (Djeriouat, 2017; Donald et al., 2021), engaging in questionable actions for financial benefits and materialistic gains. Indeed, there is a breadth of evidence linking extrinsic values to self-centeredness and competitiveness (Salazar-Ayala et al., 2021), selfishness (Sheldon & McGregor, 2000), unethical beliefs (Chowdhury & Fernando, 2013), and moral flexibility (Z. Liu et al., 2022). Experimental research has further established a link between materialistic values and the intention to act unethically. Moldes and Ku (2020) meta-analytically reviewed 27 independent studies and 62 effect sizes (N = 3649) of experimental evidence on materialism’s effect on societal well-being (i.e., factors that contribute to the positive functioning of a group) and found that materialistic values were causally and positively related to condonement of greed, stealing, and endorsement of business corruption (Moldes & Ku, 2020).

In addition, extrinsic values are closely related to the self-enhancement values in Schwartz’s (2012) theory of basic human values (Grouzet et al., 2005; Maio et al., 2009). The latter denotes the motivation to seek power, status, and achievement. It is thus not surprising that self-enhancement values were shown to positively predict attitude towards unethicality in Feldman et al.’s (2015) meta-analysis of 12 studies based on a total sample of 105,928 individuals.

Notably, the existing empirical evidence mainly comprises correlational studies (e.g., Chowdhury & Fernando, 2013) and experimental research examining attitudes or intentions toward unethical behaviours (e.g., Moldes & Ku, 2020). However, it remains unclear whether these value-directed intentions will translate into actual unethical behaviours. A recent exception is Yue et al.’s (2024) work, which found that Chinese students were more likely to lie about their performance to earn additional reward money after being primed with money importance (Experiments 2 and 3). Despite the close association between money importance and extrinsic values, it is important to restate that extrinsic/materialistic values in SDT are conceptualised more broadly, encompassing also desires for luxury goods, images, and social status that arguably better align with the focus and expectation of today’s consumption-driven societies (Kasser, 2016). This theoretical and empirical gap in literature motivated us to utilise established experimental paradigms to investigate the proposed link between extrinsic values and unethical behaviour.

In addition, SDT posits that many of its core variables and propositions are universal (Ryan & Deci, 2017). Evidence from a cross-cultural study of life values across 15 societies showed that these values were not only widely recognised and understood but also consistently organised within a similar circumplex structure (Grouzet et al., 2005). SDT further predicts that such values are linked to psychosocial processes and outcomes in comparable ways across cultures. Supporting this view, recent meta-analyses have consistently demonstrated that extrinsic values are negatively associated with a range of well-being indicators, with this relationship universally mediated by lower satisfaction of basic psychological needs (Bradshaw et al., 2023; Dittmar et al., 2014; Moldes & Ku, 2020). On this basis, we propose that the association between extrinsic values and unethicality is likewise culture-independent—a hypothesis that, to date, has not been systematically tested in the literature.

Hence, the current research collected data from Macao—a Special Administrative Region (SAR) of China—and the UK. These two societies share comparable economic and consumer-oriented profiles, both classified by the World Bank as high-income economies with high household consumption expenditure (The World Bank, n.d.), marked income inequality (CIA The World Factbook, n.d.), and strong integration into the global economy (KOF Swiss Economic Institute, n.d.). At the same time, they differ in cultural heritage, offering a valuable context to test whether the proposed relationships hold across distinct cultural settings.

We advanced two hypotheses: (1) that stronger extrinsic values would be associated with a higher likelihood of engaging in unethical decision-making and behaviour, specifically lying for financial gain, and (2) that this association would be consistent across both Macao and the UK. To test these propositions, three studies were conducted: Study 1 employed a cross-sectional, cross-country survey to examine correlations between extrinsic values and self-reported unethical decision-making, while Studies 2 and 3 used experimental paradigms conducted in laboratory and online settings to test the causal effects of exposure to extrinsic cues on lying behaviour.

## 2. Study One: Extrinsic Values and Self-Reported Unethical Decision-Making

### 2.1. Method

#### 2.1.1. Participants

Given the exploratory nature of the study, no sample size estimate was made; instead, we aimed to collect as large a sample as resources allowed. Participants were eligible to take part if they were university students aged 18 or above and able to understand the language in which the questionnaire was administered (Chinese in Macao, English in the UK). No additional exclusion criteria were applied.

In Macao, trained research assistants invited 600 university students to participate in a short questionnaire study. Participation was voluntary, with no financial incentives. Participants received an information sheet outlining the study’s aims and their rights, then gave consent by initialling a form. Five hundred and seventy-five students participated, but nine were excluded for leaving more than half of the questions unanswered, resulting in a final sample of 566 Chinese students (56.89% women; *M*_age_ = 20.36, *SD* = 1.61).

In the UK, 600 participants were recruited via the crowd-sourcing platform Prolific (www.prolific.co) for £0.80 (US$0.96). Study advertisements explained the aims, and clicking the link directed participants to Qualtrics, the online data collection platform. They read an information sheet, completed an informed consent form, and began the study. Due to Prolific’s procedures, 605 participants (68.60% women; *M*_age_ = 25.54, *SD* = 8.06) completed the survey and were all included in the analyses.

The study procedure received ethical approval from the Faculty Research Ethics Committee at the corresponding author’s institution for the UK component, and from the Psychology Department Ethics Committee of a public university in Macau for the Macanese component.

#### 2.1.2. Measures

***Extrinsic relative to intrinsic values (E/I).*** Participants rated the importance of three intrinsic values: self-acceptance (e.g., “*knowing and accepting who you really are*”), affiliation (e.g., *“having good friends that you can count on”*), and community contributions (e.g., *‘helping people in need’*) and three extrinsic values: image (e.g., *“keeping up with fashion in hair and clothing”*), popularity (e.g., *“being famous”*), and financial success (e.g., *“having a job that pays well”*) on a scale from 1 (*not at all important*) to 5 (*very important*). Each domain had three items, resulting in an 18-item scale. The Chinese version was translated from English to Chinese by Ku and Zaroff (2014) and used with university students in Hong Kong with good reliability.

Internal reliability was good for both intrinsic and extrinsic values among the UK sample, *α* = 0.72 and 0.84, respectively. But for the Chinese sample, one of the items from intrinsic values (‘*donating time or money to charity*’) had particularly low item-total correlation (*r* = 0.21) and hence were removed. Cronbach alpha coefficient was 0.70 for the intrinsic values and 0.78 for the extrinsic values after item-removal.

Kasser and Ryan (1996) recommended that it is necessary to control for the overall importance rating of values when using the Aspiration Index. This use of relative centrality score for extrinsic/intrinsic values had the advantage of considering a person’s overall value prioritisation, making it a better estimator of well-being and ill-being than simple scores of extrinsic or intrinsic values (Bradshaw et al., 2023). Putting this consideration in place, we followed procedures outlined by Duriez et al. (2007) to transform the six domains into a single extrinsic relative to intrinsic (E/I) values score. We first subtracted an individual’s overall mean score from each item score, and then reversed the intrinsic items. An overall E/I values score was then computed by averaging the extrinsic and the (reversed) intrinsic items. The internal reliability of this composite scale was good for the UK samples, *α* = 0.79 (*M* = −0.71, *SD* = 0.38), and acceptable for the Chinese ones, *α* = 0.69 (*M* = −0.55, *SD* = 0.30). Higher scores indicate higher relative importance of extrinsic values in relation to intrinsic values.

***Unethical decision-making scale.*** Detert et al. (2008) developed and validated a scale with eight ethically charged scenarios measuring participants’ willingness to engage in unethical behaviours like lying and stealing. A sample scenario was “*You work in a fast-food restaurant downtown [City X.] It’s against policy to eat food without paying for it. You came straight from classes and are therefore hungry. Your supervisor isn’t around, so you make something for yourself and eat it without paying*.” Participants rated their likelihood of engaging in each unethical behaviour on a 7-point scale ranging from 0 (*not at all likely*) to 6 = (*highly likely*). The scenarios were translated, back-translated, and pilot tested with Chinese students before the study in Macao. Due to an error in the study set-up, UK participants only answered seven scenarios. Cronbach’s *α* was acceptable for the UK sample (0.68, *M* = 3.40, *SD* = 0.74), and good for the Chinese sample (0.74, *M* = 3.15, *SD* = 0.95)^1^.

### 2.2. Results

E/I was positively correlated with unethical decision-making scale in both Macao, *r* = 0.18, *p* < 0.001, 95% CI [ 0.10–0.26], and the UK, *r* = 0.24, *p* < 0.001, 95% CI [0.16–0.31]. To compare the strength of the relationships in the two samples, we conducted Fisher’s *r* to *z* transformation and compared the two *r*s by a z-test. Results showed that the difference was not significant, *z* = 1.02, *p* = 0.309. Hierarchical regressions controlling for age (Macao: *b* = −0.03, *p* = 0.155; UK: *b* = −0.02, *p* < 0.001) and gender (Macao: *b* = 0.42, *p* < 0.001; UK: *b* = 0.05, *p* = 0.452) in Step 1 (Macao: ∆*F*(2, 559) = 15.53, *p* < 0.001, ∆*R*^2^ = 0.05; UK: ∆*F*(2, 602) = 19.96, *p* < 0.001, ∆*R*^2^ = 0.06) did not change the significant relationship between E/I and unethicality in either society in Step 2 (Macao: ∆*F*(1, 558) = 13.86, *p* < 0.001, ∆*R*^2^ = 0.02; UK: ∆*F*(1, 601) = 22.66, *p* < 0.001, ∆*R*^2^ = 0.03). In the final models, E/I significantly predicted unethicality in both Macao (*b* = 0.48, *p* < 0.001, 95% CI [0.23–0.74]) and UK (*b* = 0.37, *SE* = 0.08, *p* < 0.001, 95% CI [0.22–0.53]). These results supported the hypotheses that the relative importance of extrinsic values was positively associated with unethical decision making (H1), and that this relationship was comparable in the two samples (H2), suggesting that culture was not a factor that influenced the link.

## 3. Study Two: Causal Effect of Extrinsic Cues on Lying in MACAO

In this study, we sought to manipulate the amount of extrinsic and materialistic cues individuals may receive and examine their causal impact on actual unethical behaviour. Previous research indicates that exposure to materialistic cues in the environment, such as streets lined with luxury shops, can decrease individuals’ willingness to help others (Lamy et al., 2016). In a similar vein, Chilton et al. (2012) found that briefly prompting individuals with strong extrinsic orientations to reflect on intrinsic values temporarily heightened their concern for social justice and environmental issues. Although such effects may not reflect enduring dispositional change, they demonstrate how situational cues linked to specific value orientations can activate compatible behaviours.

Due to social desirability concerns, people may under report unethical behaviours on self-reported measures, potentially masking the true extent of unethical conduct. Experimental paradigms have been developed to mitigate this bias by offering participants the opportunity to cheat for financial gain without fear of retribution (for reviews, see Gerlach et al., 2019; Jacobsen et al., 2018). In this study, we employed the commonly used matrix task and measure lying in the laboratory (cf. Gerlach & Teodorescu, 2022). We predicted that participants who received extrinsic-cues manipulation would lie more than those who received intrinsic-cues manipulation or no-cues manipulation. As it was tangential to the main aim of our research, we did not hypothesise about the difference between the intrinsic-cues manipulation and the control condition.

### 3.1. Method

#### 3.1.1. Participants

One hundred and seventy students (*M*_age_ = 18.98, *SD* = 1.21; 115 women and 55 men) in a large public university in Macao participated in the study in exchange for research participation credits. The study was approved by the Psychology Department’s ethics committee in the university for research participation, and informed consent was obtained from all participants in the same way as described in Section 2.1.1 (for Macau participants).

#### 3.1.2. Design, Procedure, Materials and Measures

The experiment employed a between-participant design, with value-linked cues as the independent variable having three levels (no values-cues control condition, *n* = 43; extrinsic-cues condition, *n* = 43; intrinsic-cues condition, *n* = 42). Additionally, to assess actual performance, we randomly assigned 42 participants to an additional condition where lying was not possible.^2^

Each experimental session included two to four participants and lasted about 30 min. Participants read a folder with various topics, wrote a short essay based on the folder’s content, and worked on ten matrices. They could win up to MOP50 (about US$6) based on their matrix performance. After providing informed consent, participants were given a folder corresponding to their randomly assigned condition, containing photos and articles as cues for relevant values.

***Extrinsic-cues condition.*** The folder materials emphasised extrinsic values, featuring articles and photos of celebrities’ lifestyle, fashion and beauty products, and luxury dining and travel destinations.

***Intrinsic-cues condition.*** The folder materials emphasised intrinsic values, featuring articles about charitable organisations and blogs by local celebrities that highlighted self-acceptance, affiliation, and altruism.

**Figure 1:**
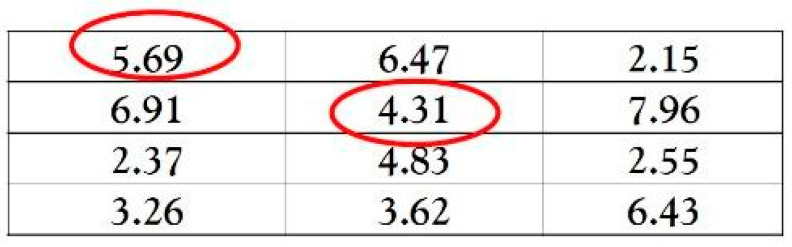
Sample of the add-to-10 matrices used in Study 2.

After writing their essays about their choices of photos and articles, participants moved to a second, ostensibly separate matrix task. They received a test sheet with 10 matrices and an answer sheet (see Figure 1). Each matrix had 12 three-digit numbers, and participants had three minutes to find two numbers that added up to 10. They were told they would earn MOP5 (about US$0.6) for each correctly solved matrix. Participants self-checked their answers and recorded the total number of correctly solved matrices on an answer sheet without experimenter verification. They folded the test sheet and placed it in their belongings, allowing them to lie without consequence (cf. Gerlach & Teodorescu, 2022).

***Control conditions.*** There were two control conditions. The *Control–Lying* condition followed the same procedure as the two experimental conditions, except that participants did not receive any value-linked cues. Instead, they read folders containing humorous stories and photos of animals and people. To accurately assess task performance, a second control condition—the *Control–No Lying* condition—was included, in which lying was not possible. Participants received no value-relevant cues and were given folders containing funny stories and photos. After the matrix task, the experimenter verified and recorded the correct answers. If participants in the extrinsic-cues condition reported significantly more correct answers than *both* of these two control conditions, it would suggest that extrinsic cues influence unethical behaviour.

At the end of each session, participants were paid based on the number of correctly solved matrices they claimed (or actually solved in the “Control-No-lying” condition) and were thanked for their participation. After all sessions, participants received a written debriefing letter explaining the study’s purpose, hypotheses, and condition designs.

### 3.2. Results

**Figure 2:**
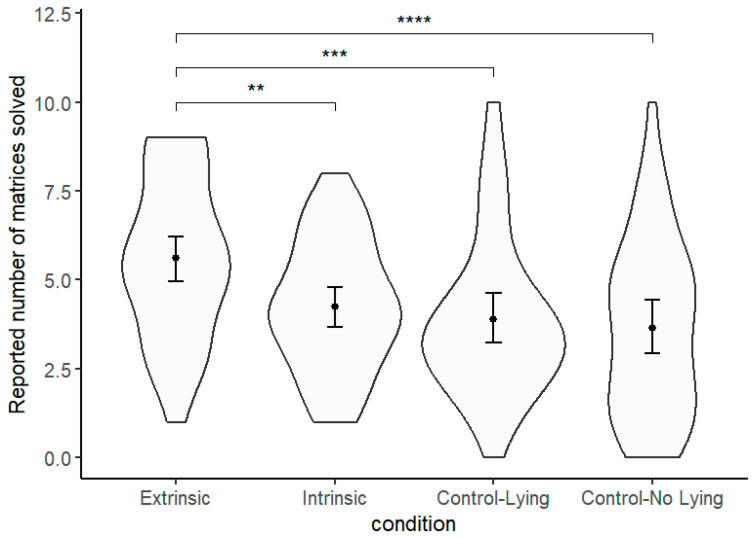
Violin plot for the reported number of matrices solved across conditions in Study 2. Points indicate condition means. Error bars indicate 95% confidence intervals. ** *p* < 0.01; *** *p* < 0.001; **** *p* < 0.0001.

Figure 2 depicts the results. An overall analysis of variance (ANOVA) revealed a significant effect of conditions on the number of correctly solved matrices, *F*(3, 166) = 6.78, *p* < 0.001, *η_p_*^2^ = 0.11. Planned contrasts showed that it was the extrinsic-cues group that differed from all the other groups, claiming to have correctly solved 5.60 matrices (*SD* = 2.24, 95% CI [4.91–6.29]), which was significantly higher than the intrinsic-cues group’s 4.24 (*SD* = 1.83, 95% CI [3.67–4.81]), *F*(1, 166) = 8.19, *p* = 0.005, *η_p_*^2^ = 0.05, the control ‘lying’ group’s 3.88 (*SD* = 2.21, 95% CI [3.20–4.56]), *F*(1, 166) = 13.15, *p* < 0.001 *η_p_*^2^ = 0.07, and the control ‘no-lying’ group’s 3.64 (*SD* = 2.48, 95% CI [2.87–4.41]), *F*(1, 166) = 16.88, *p* < 0.001, *η_p_*^2^ = 0.09.

Though it was not of primary interest, we noted from the comparisons that both the intrinsic-cues and the ‘lying’ control conditions were not significantly different from the ‘no-lying’ control condition (*p*s > 0.10) wherein actual performance was recorded, suggesting that overall participants behaved honestly. These findings were consistent with the bulk of evidence that individuals tend to be honest in the matrix task (Gerlach & Teodorescu, 2022).

## 4. Study Three: Causal Effect of Extrinsic-Cues on Lying in UK

Study 3 employed an online UK sample to assess the generalisability of the link between cuing extrinsic values and unethical behaviour. Conducting the study online was intentional, given the prevalence of online fraud and related crimes (Anderson et al., 2013). The study measured unethicality using a modified version of the ‘Die-under-a-cup’ experimental paradigm (Fischbacher & Föllmi-Heusi, 2013; Gächter & Schulz, 2016). In the original setup, participants roll a 6-sided die under a cup to conceal the outcome, then report it to the experimenter to determine their monetary payoff. Since researchers cannot verify individual participants’ honesty, and this is made clear to the participants, unethicality is inferred by comparing reported outcomes to a known statistical distribution (Moshagen & Hilbig, 2017). For example, the probability of complete honesty using a fair six-sided die is 1/6 or 16.7%.

The die-roll paradigm offers advantages over the matrix task as the outcomes are entirely random, avoiding confounding factors like participants’ desire to appear competent (Gerlach et al., 2019) or genuine reporting mistakes (Heyman et al., 2020). A meta-analysis comparing behavioural measures of unethical behaviour found that people lied significantly more in the die-roll task than in the matrix task, suggesting it may be a more sensitive measure of unethicality (Gerlach et al., 2019). Furthermore, as this is the first study to connect situational cues related to extrinsic values with unethical behaviour, we also aim to examine the generalisability of this relationship. Consistent findings with Study 2 would strengthen the conclusion that the impact of these extrinsic cues on unethical behaviour is not limited to specific research paradigms, settings, or cultures.

### 4.1. Method

#### 4.1.1. Participants

The study procedure was approved by the faculty research ethics committee of the corresponding author’s institute. All informed consent was obtained from participants before the start of the study in the same way as described in Section 2.1.1 (for UK participants).

#### 4.1.2. Design, Procedure, Materials and Measures

Given resource constraints, the directional nature of our hypothesis, and the clear findings from Study 2, we considered it appropriate to use a one-tailed test in the present study. Power analysis using G*Power 3 (Faul et al., 2007) suggested that 156 participants were required to detect a medium effect size (Cohen, 1988) with a power of 0.80 for a one-tail *t*-test at a significance level of 0.05. To allow for potential data removal due to suspicions and manipulation checks, we aimed to recruit approximately 200 participants from the UK via Prolific for £1 (US$1.24). A total of 208 individuals completed the study, but 11 expressed suspicion and were excluded. No participants were removed due to manipulation checks, resulting in a final sample of 197 participants (106 women, 89 men, 2 missing information, *M*_age_ = 37.06, *SD* = 11.83).

The study, framed as evaluating the effects of advertisements, included a control condition (*n* = 96) where participants viewed 10 abstract patterns and an experimental condition (*n* = 101) where participants viewed 10 luxury consumer product advertisements similar to those in Study 2. Each photo was shown for 10 s, followed by two questions about its effectiveness in a marketing campaign; these served to reinforce the cover story and were not included in the data analysis. Participants then answered a manipulation check question regarding how much they agreed that the visual cues promoted extrinsic values (1 = *Strongly disagree*, 7 = *Strongly agree*).

Afterwards, participants engaged in a modified multi-round die-roll task (Schindler & Pfattheicher, 2017), aiming to measure dishonesty gradients. They rolled a six-sided die ten times, using Google Dice (link provided), another online program, or a physical die nearby. Each time they rolled a “4”, they received £0.10 (US$0.12) as an extra bonus, up to a maximum of £1 (US$1.20). The expected value for honest behaviour was calculated as 16.7% × 10 = 1.67. Participants then answered a suspicion check question about the study’s purpose, with responses indicating suspicion that the study was about lying/(dis)honesty and/or (un)ethicality removed from analyses. All participants were debriefed and received the £1 bonus regardless of their die-rolling outcomes.

### 4.2. Results

Manipulation check revealed that the photos in the experimental condition group (*M* = 4.17, *SD* = 0.95) were judged as promoting extrinsic values more than the photos in the control group (*M* = 1.99, *SD* = 0.76), *t*(195) = 17.71, *p_one-tailed_* < 0.001, *d* = 2.53. Lying behaviour was inferred by comparing the group means against the statistical baseline of 1.67. Both the experimental group (*M* = 2.70, *t*(100) = 4.57, *p* < 0.001, *d* = 0.45) and the control group (*M* = 2.25, *t*(95) = 4.15, *p* < 0.001, *d* = 0.42) reported significantly more rolled “4s” than the expected frequency of 1.67, indicating the *occurrence* of lying in both conditions. One-tailed independent sample *t*-test was then performed on the difference score between the number of rolled ‘4’ and the baseline to compare the *magnitude* of lying between the experimental and control conditions. Results indicated that the experimental group (*M* = 1.03, *SD* = 2.27, 95% CI [0.58–1.48]) lied to a greater extent than the control group (*M* = 0.58, *SD* = 1.37, 95% CI [0.30–0.85]), *t*(165.47) = 1.70, *p_one-tailed_* = 0.045, *d* = 0.24, consistent with the findings of Study 2 that when individuals received visual cues related to extrinsic values, they were more likely to lie for financial gains.

## 5. Discussion

Across three studies, we tested the idea that extrinsic values are positively related to unethicality. Study 1 cross-sectionally demonstrated in Macao and the UK that those who were relatively more extrinsically aspirated made more unethical decisions in hypothetical scenarios. These results converged with actual behavioural measures in a lab-based experiment amongst Macao university students (Study 2), as well as in an online experiment amongst UK-based online workers (Study 3). Specifically, participants overstated their performance in a matrix task (Study 2) and over-reported the times they rolled a target number (Study 3) in exchange for extra bonuses after receiving extrinsic cues. The studies thereby provided consistent evidence to support the notion that extrinsic values are related to unethical behaviour, regardless of the methods, samples, measurements, and settings of the investigations.

These findings extend the literature pertaining to the detrimental effects of extrinsic aspiration. While there is a wealth of research showing that materialistic values are associated with personal ill-being such as poorer physical and mental health (Dittmar et al., 2014), much less attention has been given to their impact on societal well-being (Moldes & Ku, 2020). Yet, the benefits of a healthy and community-based society cannot be understated as societal conditions are key determinants of citizens’ personal wellness (for an overview, see Veenhoven, 2015). The current research thus adds to the emerging body of research concerning societal well-being issues caused by extrinsic and materialistic pursuit (e.g., Yue et al., 2024). Specifically, our studies suggest that such pursuit could instigate unethical behaviour, which potentially causes further societal damages like corruption and distrust (Spadaro et al., 2022; Tang et al., 2023) that may recursively undermine personal well-being (Tay et al., 2014).

Of particular importance are the results of Studies 2 and 3 that being exposed to situational cues that are linked to extrinsic values could casually increase participants’ tendency to lie. If participants could temporarily become less ethical just by being exposed to extrinsic cues, the constant bombardment of materialistic messages and ideals in the media and advertising may perpetually activate people’s extrinsic values, leading to the normalisation of unethical practices and illegal activities. But there is a silver lining: if situational extrinsic cues can momentarily increase the proclivity to act dishonestly; conversely, the activation of intrinsic, community-oriented values should strengthen people’s adherence to social norms and concerns for others whilst inhibit opposing materialistic pursuits and corresponding ethically incompatible behaviours (Kasser, 2016; Maio et al., 2009). However, we were not able to demonstrate this through Study 2 as a floor effect was observed: those who received intrinsic-linked cues were not significantly different from the two control groups in their behaviour, as it seemed there was very little to no lying going on in these three groups. Nevertheless, past research has demonstrated the benefits of intrinsic values intervention, such as enhancing individuals’ self-concepts (Cheon et al., 2019), encouraging learning motivation and outcomes (Froiland, 2018; Ku et al., 2022a), as well as improving young people’s body esteem (Ku et al., 2022b). Drawing data from 49 countries, Kremer (2024) further illustrated that, upon accounting for per capita incomes, a focus on intrinsic values at the national level correlates positively with the countries’ reported levels of life satisfaction and happiness. Together, these findings suggest that strengthening the emphasis on intrinsic values, both at the individual and national levels, could be advantageous and should be integrated into national policies and international development cooperation efforts. Future research could use cross-level analyses to evaluate the effectiveness of such intervention.

Another important finding from the current research is that the link between extrinsic values and unethical behaviour appeared to be similar in the Macao and UK samples. These corroborate the findings that self-enhancement values are associated with unethicality (Feldman et al., 2015) and individualising foundations (Feldman, 2021) but culture did not moderate their relations. Taken together, these findings are aligned with SDT’s universality principle (Ryan & Deci, 2017) and suggest that there may exist a basic, universal link between extrinsic values and unethicality. This can be explained in part by the fact that values play an important role in shaping human’s sensemaking processes. That includes the interpretation of how urgent and valuable something is, and the judgement of what behaviours are necessary and appropriate to attain it (Sagiv & Roccas, 2021). Since extrinsic aspiration is related to self-serving moral judgments (Z. Liu et al., 2022; Sheldon et al., 2018), those who received extrinsic cues in our Macao and UK samples might have undergone similar reasoning and resulted in higher incidence of unethical actions. Furthermore, the value-unethicality link we found may reflect the rise of highly competitive and market-driven economies worldwide (Gwartney et al., 2022) that is independent of a society’s cultural heritage and norms (Hamamura, 2012). Indeed, despite differences in sociocultural background, both Macao and the UK are capitalistic economies that experience a high level of economic inequality (CIA The World Factbook, n.d.). Previous research has shown that this economic system is linked to higher acceptance of inequality (Piketty, 2015) and greed (Greenfeld, 2009). These factors may override the prescriptive norms within a culture to regulate people’s extrinsic pursuit.

The current findings contribute to two streams of literature that could synergise each other. The first is the literature related to the motivation behind everyday dishonesty, which has been explained through the lens of morality such as the self-concept maintenance theory and the moral disengagement theory (see Jacobsen et al., 2018, for a review). We, on the other hand, link unethical/dishonest behaviour to extrinsic/materialistic values that are not inherently linked to morality. The advantage of this approach is that, unlike morality which is contextually bounded (Graham et al., 2016), the influences of values are relatively stable and trans-situational (Schwartz, 2012). That means researchers can easily generate hypotheses about who, when, and why people behave unethically across research contexts. Additionally, a value-based investigation offers a different explanation for why some people are dispositionally honest or dishonest (Hilbig, 2022)—perhaps because they pursue different aspirations.

Second, we contribute to the literature on SDT by extending its application to societal well-being. Previous studies on SDT, especially the ones based on GCT, have mostly focused on the distinct effects of intrinsic and extrinsic values on the satisfaction of basic human needs (Ryan et al., 2022; Ryan & Deci, 2019). Individual flourishing as a result of self-determination is the pivot to this line of research; it has been widely studied in various domains including healthcare, education, organisations, and technology (Ryan & Deci, 2019). Relatively fewer studies have taken societal well-being or ill-being as the end-outcome of an individual’s (lack of) self-determination and endorsement of extrinsic/intrinsic life values (Moldes & Ku, 2020). To illustrate, SDT has been applied to topics such as pro-environmental attitudes and behaviour (Ku & Zaroff, 2014; Masson & Otto, 2021) and ethnic/racial prejudice (Duriez et al., 2007). Many of these behaviours can be considered as morally relevant because they entail responses to the needs and interests of others (Eisenberg et al., 2013). Surprisingly, it is only until recently that theoretical and empirical work integrating moral motivation and behaviour into the SDT framework has been seen (Curren & Ryan, 2020; Donald et al., 2021). A relevant example was the research conducted by Sheldon et al. (2018), which revealed across three studies that the combination of extrinsic values and extrinsic reasoning consistently predicted immoral behaviour. The researchers explained their results in light of participants feeling being socially controlled in the pursuit of extrinsic contingencies, thereby removing the sense of responsibility for their own unethical action. We extend their results by showing that the exposure to extrinsic cues (Studies 2 and 3) could casually increase dishonest behaviour across two cultural samples. In future studies, it may be beneficial to combine another mini-theory, the Organismic Integration Theory, which directly addresses the distinction between extrinsic and intrinsic motivations (Ryan & Vansteenkiste, 2023), with Goal Contents Theory. This approach would involve manipulating not only values content but also behavioural motivations.

## 6. Limitations and Directions for Future Research

Several limitations should be noted when interpreting the present findings. First, although we found consistent evidence that exposure to cues linked to extrinsic values predicted actual unethical behaviour in the matrix (Study 2) and die-roll tasks (Study 3), these paradigms were designed such that participants could report their outcomes with impunity. The strength of this design is that it precludes the concern about social desirability bias; however, this also makes it impossible to determine who actually lied at the individual level. The degree of dishonesty could only be inferred either by comparing the extrinsic-cues condition with the control, as in Study 2, or estimating it from a statistical baseline, as in Study 3. As a result, the current designs were not best-suited for empirically examining the mechanisms that underlies the association between extrinsic cues and unethicality, even though potential mediators have been proposed in the literature, including interpersonal competitiveness (Bauer et al., 2012), moral disengagement (Sheldon et al., 2018), and moral flexibility (Z. Liu et al., 2022). Future research could use (modified) paradigms to assess actual dishonest behaviour at the individual level (J. Liu et al., 2021; Pascual-Ezama et al., 2020) and substantiate our findings with tests of mediation.

Another limitation of Studies 2 and 3 is that we did not examine potential interactions between participants’ dispositional values and the situational cues to which they were exposed. Individual value orientations may shape how people interpret and respond to specific cues (Sagiv & Roccas, 2021), suggesting that the effects of extrinsic versus intrinsic cues might vary depending on participants’ pre-existing values. Indeed, recent work highlights growing interest in the interactive—rather than merely additive—roles of dispositional and situational factors in predicting dishonest behaviour (Hilbig, 2022). Future research should therefore test such person–situation interactions to provide a more comprehensive understanding of how life values influence unethicality.

The generalisability of the results is limited in three respects. First, while unethical behaviour was assessed in various forms, the measures mainly focused on everyday lying for personal gains, raising questions about whether extrinsic aspiration similarly motivates dishonest behaviour without financial incentives (Cantarero et al., 2018).

Second, the study used samples from Macao and the UK, both of which are developed capitalist economies. Individuals in such contexts may differ in their motivational priorities from those in less developed economies, where aspirations for money and possessions often serve basic needs for security and survival (Brdar et al., 2009). Future research comparing cultures at different stages of economic development would therefore be valuable in enhancing the cross-cultural applicability of SDT to understanding unethical behaviour. Moreover, although the present research proposes that unethical behaviour is linked to extrinsic values regardless of broader cultural orientations such as individualism and collectivism, the horizontal and vertical dimensions of these cultural patterns (Oyserman et al., 2002; Triandis & Gelfand, 1998) may nonetheless influence how extrinsic values relate to unethical behaviour. For instance, vertical individualism—a cultural pattern characterised by competition, social comparison, and acceptance of inequality—tends to reinforce extrinsic value pursuits such as status and material success. This orientation aligns closely with what Kasser et al. (2007) described as the ethos of *American corporate capitalism*, in which individuals are encouraged to equate personal worth with financial achievement and outward success. Such cultural climates may normalise or even legitimise ethically questionable behaviour in the pursuit of competitive advantage. In contrast, more horizontal cultures, which emphasise equality, may mitigate these tendencies by valuing cooperation and community well-being over self-enhancement. Understanding how these broader cultural dimensions interact with value orientations would provide deeper insight into the motivational roots of unethical decision-making across societies.

Third, while the study procedures, participation fees, and investigation contexts differed between the UK and Macao samples in Study 1, as well as between Studies 2 and 3, these variations necessarily limit the possibility of direct comparison. However, it is noteworthy that despite these methodological differences, the findings across countries and study designs were largely comparable. This consistency suggests that the association between extrinsic values and unethical decision-making may reflect a general psychological mechanism rather than a context-specific effect. For the two experiments in particular, this limitation also represents a methodological trade-off: while the differing experimental designs preclude direct comparison, they provide convergent evidence that the link between extrinsic values and unethical behaviour is not confined to a specific design or method. Nevertheless, future research should conduct direct, preregistered replications to confirm the robustness of these causal effects. In particular, variations in experimental design, setting, and the framing of incentives should be systematically examined to further clarify the generalisability of the observed effects across different methodological and cultural conditions.

## 7. Conclusions

Drawing on self-determination theory, this paper reports evidence from two surveys and two experiments conducted in culturally distinct societies, showing that extrinsic/materialistic values are positively associated with unethical behaviour. This relationship appears consistent across cultures and robust to variations in measurement and context. These findings highlight the growing concern that extrinsic aspirations may carry detrimental consequences for societal well-being.

## Data Availability

All related materials and data, including questionnaires, experimental manipulation materials, data and syntax files are available from OSF: https://osf.io/pfrw8/?view_only=d8ea2c20baab47f3a6c93bf723f01c94.

## Notes

1 Analyses for the Chinese sample were conducted using the full 8-item scale. Utilising a 7-item scale for both samples yielded consistent results. Further details are provided in the syntaxes available on OSF (https://osf.io/pfrw8/?view_only=a63feec2da22420f889f99095384a704).

2 We did not conduct a prior power calculation for this study, but a post hoc sensitivity analysis using G*Power (Faul et al., 2007) suggested that our sample size of 170 participants had the power of 80% (α = 0.05) to detect a minimum effect size of *f* = 0.26 or *η_p_*^2^ = 0.06 (i.e., a medium effect size) for the key main effect of condition, which was below our observed effect of *η_p_*^2^ = 0.11.
